# Studies Raise Questions about Pavement Sealers

**DOI:** 10.1289/ehp.120-a192a

**Published:** 2012-05-01

**Authors:** Bob Weinhold

**Affiliations:** Bob Weinhold, MA, has covered environmental health issues for numerous outlets since 1996. He is a member of the Society of Environmental Journalists.

Airborne emissions and stray dust from coal tar–based sealers, one of the two main types of products used to coat certain asphalt pavements, may be a more significant human health threat than previously thought, according to three new studies and a review published by U.S. government and university researchers.^1^^,^^2^^,^^3^^,^^4^ The findings build on previous research on coal tar–based sealers (mostly on environmental impacts via runoff and other pathways) and provide novel insights on pathways of human exposure and the magnitude and duration of the emissions. But “we’re just at the very beginning as far as [understanding] human health effects,” says Peter Van Metre, coauthor of all four publications and a research hydrologist with the U.S. Geological Survey.

Asphalt is used extensively to pave parking lots, driveways, airport runways, roads, playgrounds, paths, and other surfaces. Sealers are marketed as products that can help prevent pavement degradation and improve appearance, and they are used across the United States on all types of asphalt surfaces, with the exception of roads. The two most popular sealer types are emulsions based on either refined coal tar or asphalt ingredients.^5^

Coal tar–based sealers contain an average concentration of 16 polycyclic aromatic hydrocarbons (PAHs) about 1,300 times greater than that found in asphalt-based sealers,^4^ and in 2003 coal tar–based sealers became a suspect contributor to the elevated concentrations of PAHs in metropolitan areas.^6^ PAHs are known or suspected to cause cancer, reproductive problems, birth defects, genetic mutations, and damage to the liver, blood, skin, and immune system, although data for both people and animals are limited.^7^ The primary source of PAHs is thought to be incomplete combustion of organic substances by sources such as power plants, vehicles, wildfires, food grilling, and cigarettes, although they also occur in a variety of consumer products.^7^

Until now, it has been widely assumed that the primary source of human PAH exposure for nonsmokers is food. But in one of the new studies, researchers calculated that ingestion of PAH-contaminated indoor dust via hand-to-mouth contact was on average 14.5 times higher for young children living in apartments next to parking lots treated with coal tar–based sealer compared with children living next to untreated parking lots.^1^ That intake made indoor dust a greater source than food by an average of 2.5 times, whereas the relatively low intake of PAHs via dust for children living next to untreated lots left food as their dominant source.

In another of the studies, conducted during a central Texas summer, researchers evaluated airborne PAH emissions from sealed and unsealed parking lots.^2^ During the hottest part of the day they found that average concentrations of eight PAHs about an inch above the surface were 19 times greater for the coal tar–treated lots than for unsealed lots, and were 5 times greater about four feet above the surface.^2^ The PAH concentrations over the sealed lots were 3.2 times greater during the heat of the day than during the coolest part of the day, and the average rate of volatilization during the day was 62 times greater for coal tar–treated lots than for unsealed lots, Van Metre says. Even a lot sealed eight years earlier produced emissions in the middle of the range, indicating the surfaces can remain a long-term PAH source.

**Figure:**
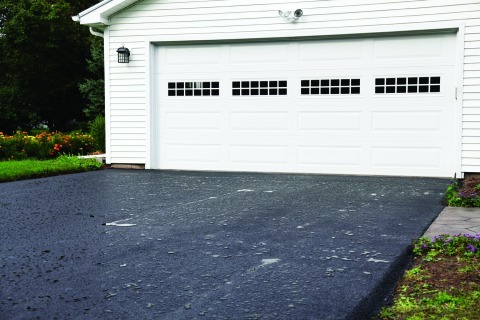
State and local jurisdictions in 10 states and the District of Columbia have imposed some restriction on the use or sale of coal tar–based sealers. © Willowpix.com/iStockphoto.com

Looking at the bigger picture, the authors estimate that, in the United States each year, the mass of emissions of six major PAHs within the first 16 days after application of all coal tar–based sealer projects may be about 20 times greater than that from all annual vehicle emissions.^8^ However, the team acknowledges its calculation could be significantly off one way or another due to many uncertainties in the factors in their equation.

Asphalt-based sealers are considered less effective and durable for some surfaces because they are less resistant to petroleum-based products, ultraviolet light, and salts than their coal tar–based counterparts, says Anne LeHuray, executive director of the Pavement Coatings Technology Council, which represents about 20 manufacturers that make both types of products. She vigorously contests conclusions that coal tar–based sealers pose any substantial health or environmental problems. However, among paving contractors, even in the same city, there can be diametrically opposing views about the performance, cost, health, and environmental pros and cons of coal tar– and asphalt-based sealers.^9^

Kent Hansen, director of engineering for the National Asphalt Pavement Association (whose members deal with the asphalt pavement protected by sealers), says he questions the need for sealers at all. “We really don’t have any strong science on the benefits,” he says, while noting that sealers can provide short-term cosmetic appeal. Adds the association’s spokeswoman, Margaret Cervarich, “The [asphalt pavement] structure should last forever,” since a quality job can have its life extended with a thin coat of repaving after about 20 years, along with interim crack sealing.

Citing the growing evidence suggesting health and environmental harm, as well as options for avoiding the use of sealers or at least using potentially less-toxic sealers such as asphalt-based products, 27 state and local jurisdictions in 10 states and the District of Columbia have imposed some restriction on the use or sale of coal tar–based sealers, and at least 7 national or regional retailers have stopped selling them.^10^

Barbara Mahler, another coauthor of all four recent publications and a research hydrologist at the U.S. Geological Survey, says she expects this fledgling field of investigation to begin gathering steam. “We’re starting to see others begin to pick up the ball [following the older environmental studies],” she says. “As this gets on people’s radar screen, we’ll start to see a lot more on human health studies.”
